# Brugada Syndrome and Pulmonary Atresia with Intact Interventricular Septum: Fortuitous Finding or New Genetic Connection?

**DOI:** 10.3390/genes15050638

**Published:** 2024-05-17

**Authors:** Miguel Fogaça-da-Mata, Estefanía Martínez-Barrios, Lorenzo Jiménez-Montañés, José Cruzalegui, Fredy Chipa-Ccasani, Andrea Greco, Sergi Cesar, Núria Díez-Escuté, Patricia Cerralbo, Irene Zschaeck, Marcos Clavero Adell, Ariadna Ayerza-Casas, Daniel Palanca-Arias, Marta López, Oscar Campuzano, Josep Brugada, Georgia Sarquella-Brugada

**Affiliations:** 1 Arrhythmia, Inherited Cardiac Diseases and Sudden Death Unit, Hospital Sant Joan de Déu, Passeig Sant Joan de Déu 2, Esplugues de Llobregat, 08950 Barcelona, Spain; mmata@ulslo.min-saude.pt (M.F.-d.-M.); estefania.martinez@sjd.es (E.M.-B.); josecarlos.cruzalegui@sjd.es (J.C.); fredy.chipa@sjd.es (F.C.-C.); andrea.greco@sjd.es (A.G.); sergi.cesar@gmail.com (S.C.); nuria.diez@sjd.es (N.D.-E.); patricia.cerralbo@sjd.es (P.C.); irene.zschaeck@sjd.es (I.Z.); 2 Pediatric Cardiology Unit, Hospital de Santa Cruz, Centro Hospitalar Lisboa Ocidental, 1449-005 Lisbon, Portugal; 3 European Reference Network for Rare, Low Prevalence and Complex Diseases of the Heart (ERN GUARD-Heart), 1105 AZ Amsterdam, The Netherlands; 4 Arrítmies Pediàtriques, Cardiologia Genètica i Mort Sobtada, Institut de Recerca Sant Joan de Déu, Santa Rosa 39–57, Esplugues de Llobregat, 08950 Barcelona, Spain; 5 Medical Science Department, School of Medicine, Universitat de Girona, 17003 Girona, Spain; oscar@brugada.org; 6 Pediatric Cardiology Unit, University Hospital Miguel Servet, 50009 Zaragoza, Spain; ljimenezmo@salud.aragon.es (L.J.-M.); mclaveroa@salud.aragon.es (M.C.A.); aayerzac@hotmail.com (A.A.-C.); danielpalanca@hotmail.com (D.P.-A.); martalpzr@gmail.com (M.L.); 7 Centro de Investigación Biomédica en Red, Enfermedades Cardiovasculares, 28029 Madrid, Spain; josep@brugada.org; 8 Cardiovascular Genetics Center, University of Girona—IDIBGI, 17190 Girona, Spain; 9 Arrhythmias Unit, Hospital Clinic de Barcelona, Universitat de Barcelona, 08036 Barcelona, Spain; 10 Department of Surgery and Medico-Surgical Specialties, School of Medicine and Health Sciences, Universitat de Barcelona, 08036 Barcelona, Spain

**Keywords:** Brugada syndrome, right ventricle outflow tract, pulmonary atresia, genetics

## Abstract

Brugada syndrome is a rare arrhythmogenic syndrome associated mainly with pathogenic variants in the *SCN5A* gene. Right ventricle outflow tract fibrosis has been reported in some cases of patients diagnosed with Brugada syndrome. Pulmonary atresia with an intact ventricular septum is characterized by the lack of a functional pulmonary valve, due to the underdevelopment of the right ventricle outflow tract. We report, for the first time, a 4-year-old boy with pulmonary atresia with an intact ventricular septum who harbored a pathogenic de novo variant in *SCN5A*, and the ajmaline test unmasked a type-1 Brugada pattern. We suggest that deleterious variants in the *SCN5A* gene could be implicated in pulmonary atresia with an intact ventricular septum embryogenesis, leading to overlapping phenotypes.

## 1. Background

Brugada syndrome (BrS) has seen riveting changes since its first description in 1992. Currently, diagnosis of BrS follows an electrocardiographic (ECG) pattern, showing an ST-segment elevation with type I morphology > 2 mm in >1 lead among the right precordial leads V1, V2 positioned in the 4th intercostal space (in the standard ECG) or the 2nd and 3rd intercostal spaces (if high parasternal leads disposal) [1]. These electrical disturbances may lead to malignant arrhythmias and even sudden cardiac death (SCD), sometimes the first symptom of the disease. The characteristic ECG pattern is a hallmark of the BrS and can be observed either spontaneously or after provocative testing with a class I antiarrhythmic drug or with an external trigger, mainly fever. BrS was initially described as a purely ion cardiac channelopathy, with no structural heart alterations despite scattered morphological and cardiomyocyte histopathological changes have been reported in some cases, showing biventricular fibrosis with increased collagen content, mostly affecting the right ventricular outflow tract (RVOT) [2]. This is the area where the highest arrhythmogenic potential has been described [3], suggesting that if these structural alterations in patients diagnosed with BrS are cause or consequence of arrhythmogenic events [4].

Nowadays, BrS is definitively linked to deleterious variants only in one gene, the *SCN5A* gene, responsible in 20–30% of cases [1]. The *SCN5A* gene (3p22.2, ID:6331) encodes for the sodium voltage-gated channel α subunit 5 (Nav1.5, MIM:600163). Recent advances in the field of genetics showed that deleterious variants in this gene have been associated with a wide spectrum of phenotypes including purely life-threatening arrhythmias, dilated cardiomyopathy, and epilepsy among others [5]. Other rare variants located in minor genes has been reported as a potential cause of BrS despite no conclusive data supports this association so far [1]. Therefore, a proper classification and interpretation of rare variants is crucial to allow for a transition of genetic data into clinical practice, helping to unravel the cause of the disease. It is especially challenging in the *SCN5A* gene because it has been related to different phenotypes. In addition, unravelling the genetic origin of the BrS also allows for cascade genetic testing in relatives, leading to early identification of family members who carry the same genetic variant(s) and are thus at risk of suffering malignant arrhythmias. The identification of genetic variants facilitates personal care to reduce the risk of sudden cardiac death, especially in young male individuals who carrier the variant.

Pulmonary atresia with intact ventricular septum (PA-IVS) is a congenital heart disease (CHD) marked by the absence of a functional pulmonary valve. It is due to altered embryogenesis culminating in an underdeveloped right ventricular chamber, and specifically affects the RVOT [6]. Long-term follow-up of PA-IVS patients has shown a high incidence of arrhythmic malignant events, with SCD being one of the causes of late mortality [6]. These can be of secondary etiology when associated with post-procedural surgical or percutaneous lesions, but in cases of overlap of CHD and arrhythmic disease (such as BrS), a primary etiology associated with an arrhythmogenic substrate can be postulated. Currently, several cases of BrS associated with pathogenic *SCN5A* variants have been implicated in overlap phenotypes with CHD, mainly ventricular septal defect [7], suggesting a common embryologic origin leading to structural and arrhythmogenic defects, despite no PA-IVS concomitant to BrS being reported to date.

## 2. Case Presentation

**Figure 1:**
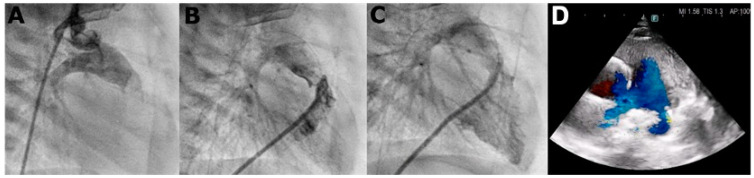
Angiography performed at the neonatal period showing: retrograde injection through the aorta showing pulmonary atresia (panel **A**), anterograde injection at the right ventricle outflow tract after perforation of the pulmonary valve (panel **B**), and final anterograde injection of the right ventricle after the final valvuloplasty (panel **C**). Echocardiography at 4 years-old showing the right ventricle outflow tract with the pulmonary arteries (panel **D**).

**Figure 2:**
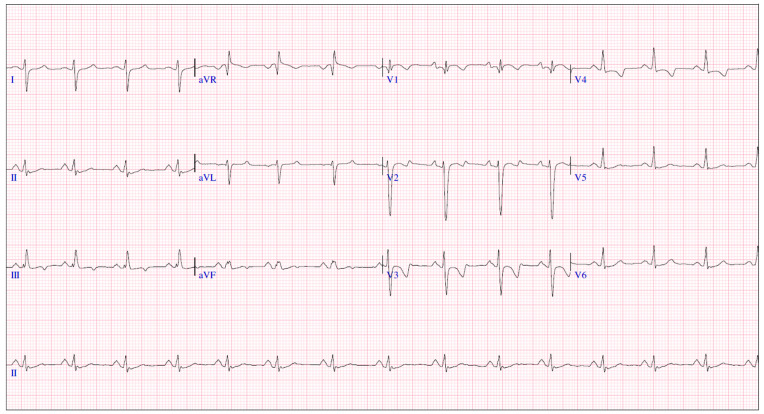
Baseline ECG.

We report a 4-year-old boy with a history that includes a prenatal diagnosis of PA-IVS (International Paediatric and Congenital Cardiac Code 01.01.07) [8] with confluent pulmonary branches. He underwent neonatal percutaneous pulmonary valvulotomy (Figure 1) with no significant RVOT gradient. No signs of coronary abnormalities were found. During follow-up, he presented signs of neurodevelopmental delay with autistic traits. For this reason, a genetic analysis through an exome TRIO identified a rare nonsense de novo variant in *SCN5A* gene (c.1603C>T, p.Arg535Ter) in heterozygosis, previously reported in patients with BrS and classified as pathogenic. Consequently, he was referred to our pediatric arrhythmia center, the reference center for rare inherited cardiac diseases. The basal ECG (Figure 2) presented sinus rhythm, PR of 154 ms, QRS duration of 88 ms, negative T waves in precordial leads V1–V4, and no Brugada ECG pattern, neither in standard nor in high precordial lead placement. The echocardiogram (ECHO) showed a dilated right atrium, moderate tricuspid regurgitation, right ventricle hypertrophy and mild dilation, and moderate pulmonary regurgitation, with no significant RVOT residual gradient nor additional vascular malformations such as aorto-pulmonary collaterals. No signs of coronary abnormalities were found during the neonatal catheterization.

**Figure 3:**
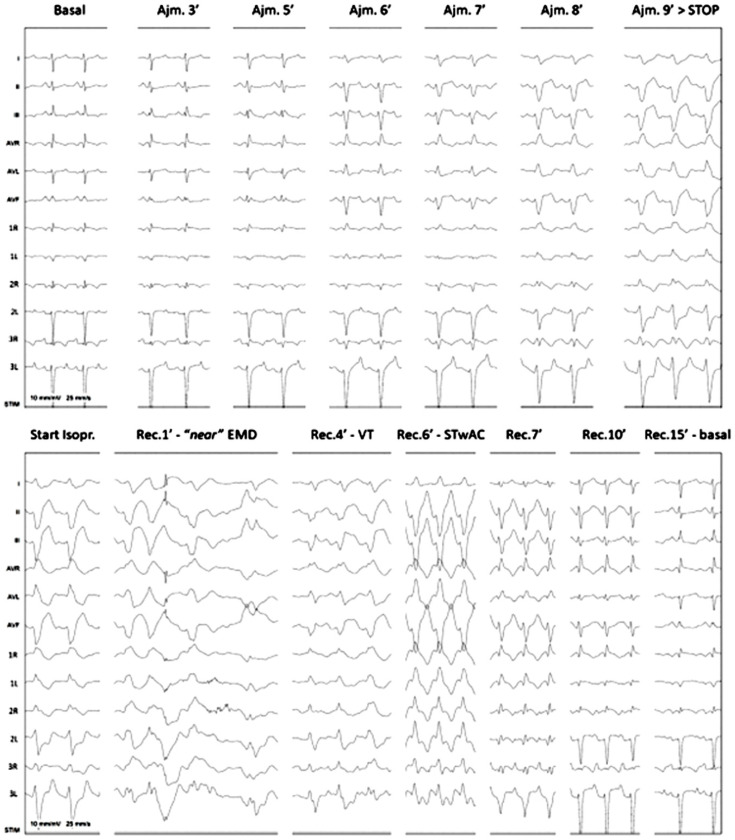
**Positive Ajmaline challenge inducing ventricular tachycardia**. Ajm—ajmaline; EMD—electromechanical dissociation; Isopr—isoproterenol; Rec.—post ajmaline recuperation; STwAC—sinus tachycardia with aberrant conduction; VT—ventricular tachycardia.

After a multicentric discussion, a diagnostic ajmaline challenge was warranted to rule out BrS at age 4 (Figure 3). Ajmaline continuous infusion at 1 mg/kg during 10 min and high precordial lead placement (1st, 2nd, and 3rd intercostal spaces at the right and left sternal borders) was performed. At the 3rd minute, subtle changes started to appear, with slight QRS enlargement and J point elevation below pathological threshold. These findings worsened and a diagnostic type 1 Brugada pattern, with J point elevation >2 mm and severe QRS enlargement, was clearly seen at the 9th minute of infusion. The infusion was stopped, but the changes progressed into ventricular tachycardia. Isoproterenol infusion was started, but the child had severely reduced cutaneous perfusion and pulse amplitude, and ECHO showed electromechanical dissociation with marked dyskinetic contraction and reduced cardiac output, with need for chest compressions for approximately 30 s. The isoproterenol infusion gradually induced sinus tachycardia with aberrant conduction, the time at which the infusion was stopped. Conduction aberrancy slowly resolved, and after normalization of the ECG, a loop recorder was implanted. This test was followed by an electrophysiological study which identified basal intervals were normal (AH 95 ms, HV 45 ms, anterograde Wenckebach point 340 ms, VA dissociation at 600 ms) as well as sinus node recovery times. Right ventricle stimulation (with the *Maastrich* protocol) did not induce ventricular arrhythmias. A diagnosis of low risk BrS was assumed based on clinical and electrophysiological data and the child was discharged the following day.

Exome TRIO genetic analysis performed from peripheral blood. Genomic DNA was analyzed using next-generation sequencing (NGS) technology, as previously reported by our group [9]. After a comprehensive bioinformatic analysis and Sanger validation, only one rare variant was identified as genetic diagnosis. This rare variant was classified following current recommendations of the American College of Medical Genetics and Genomics (ACMG) [10]. In addition, the rare variant was consulted in ClinGen (www.clinicalgenome.org), VarSome (www.varsome.com), CardioClassifier (www.cardioclassifier.org), CardioVAI (www.cardiovai.engenome.com), CardioBoost (www.cardiodb.org/cardioboost/), ClinVar (www.ncbi.nlm.nih.gov/clinvar/intro/), MasterMind (https://mastermind.genomenon.com), LitVar2 (www.ncbi.nlm.nih.gov/research/litvar2/) and Genome Aggregation Database-GnomAD- (http://gnomad.broadinstitute.org/). Finally, to avoid bias, three cardiogenetic specialists independently classified this rare variant identified and all investigators agreed to the final interpretation.

The study was approved by the Ethics Committee of the Hospital Sant Joan de Déu (Barcelona, Spain), following the Helsinki II declaration. Written informed consent to participate in this study was provided by the participants’ legal guardians. Written informed consent was also obtained from all relatives included in the study in order to perform both clinical assessment and genetic analysis.

## 3. Discussion

Deleterious variants in the *SCN5A* gene have reportedly been associated with a wide spectrum of phenotypes, mainly related to BrS, but also to other inherited arrhythmogenic syndromes. It is important to discard the named BrS phenocopies in the clinical assessment (myocardial ischaemia, electrolyte disturbances, or drug-induced and fever-induced) which may induce the characteristic pattern in the ECG, but not real BrS should be diagnosed. To our knowledge, this is the first case of BrS patient concomitant with PA-IVS in the context of a pathogenic variant in the *SCN5A* gene. The fact that both pathologies share a preferential pathophysiological location in the RVOT raises questions regarding a more than coincidental connection. Altered blood flow patterns in the embryological phase in patients with BrS and VSD have been hypothesized as possibly inducing worse RVOT fibrosis and a more severe BrS course [7]. Similarly, the question could be raised that changes in *SCN5A* could, in some cases, induce severe RVOT fibrosis in the embryological phase, ultimately conditioning PA-IVS/critical pulmonary stenosis. Moreover, SCD of arrhythmic etiology is an issue in long-term follow-up of PA-IVS patients [6], suggesting that some of these patients could have overlapping phenotypes, mainly due to deleterious variants in the *SCN5A* gene. We have to highlight the possibility that development of arrhythmias during follow-up could be secondary to the anatomic alterations, in addition to the potential malignant role of the *SCN5A* variant.

The *SCN5A* variant (c.1603C>T, p.Arg535Ter) has been previously reported (rs1417036453) (https://www.ncbi.nlm.nih.gov/snp/?term=), and is always associated with BrS and in a case of a child diagnosed with VSD at birth who was later diagnosed with BrS [7]. The alteration induced an aminoacid change in Arginine (Arg, R)—polar uncharged side chains—to a codon STOP, reducing the length of the protein by almost one quarter (in the position 535 of the 2016 aminoacids). With such a premature termination of translation, the mRNA will probably be subject to nonsense-mediated decay (NMD), leading to a decrease in protein expression [11]. This position is highly conserved between different species, highlighting the key role. This rare nonsense variant showed a current global frequency of 0.0004% (7/1461302). Family segregation showed no clinical defects in any of the close relatives, as well as no harboring of the same genetic alteration, concluding the de novo variant in the index case. The variant was currently classified as pathogenic following ACMG/AMP recommendations (PVS1, PP5, PM2, PM6) [10]. All data allows us to conclude a pathogenic role of this rare variant and the cause of the phenotype diagnosed in the index case, based on the current available data, both clinical and genetic.

The pathogenicity of the variant, as well as *the novo* identification in our index case, supports the causative role of the variant as the origin of the BrS, but also as being responsible for PA-IVS. The first published report did not show any data concerning the patient/family harboring p.R535X [12]. In 2005, this nonsense variant was reported in an asymptomatic BrS patient with a typical ECG-type 1 pattern, but only during fever; functional study of this variant supported the deleterious role in sodium current [13]. One year later, the same variant was reported in a patient with typical coved ST elevation during a pharmacological challenge [14]. In 2009, the same alteration was reported in a large family in which the largest part of genetic carriers did not basally show the ECG-type 1, but unmasked the diagnostic pattern after a pharmacological test [15]. In 2021, the same variant was identified in a case of an unexplained SCD with no prior clinical history [16]. Recently, this same variant was previously identified in the case of a patient diagnosed with VSD and BrS. In this study, the authors hypothesize that some loss-of-function variants in *SCN5A* in patients with BrS and VSD would alter blood flow in the RVOT, leading to fibrosis and electrophysiological changes, which would predispose them to an earlier clinical presentation of BrS [7]. These previous studies are in concordance to our case. It is important to mention that a comprehensive genetic analysis helps to unravel the cause of the disease in most cases. To achieve this, genetic panels that include hundreds of genes, or even complete exome sequencing, are already being carried out as part of clinical protocols.

To perform an ajmaline test poses serious risks for patient well-being, and the need to perform it in adequate conditions with proper medical support becomes obvious with our case, despite not often being life-threatening. The possible presence of a right bundle branch block pattern in PA-IVS could deter suspicions of associated BrS. In recent years, some reports suggested the oligogenic origin of BrS more than a purely monogenic disease; this point remains as a main matter of argument due to scattered BrS cases having been reported so far following this pattern of inheritance. However, the causative role of the rare nonsense variant as a cause of BrS is not in discussion to date. The potential association of BrS and PA-IVS due to the same rare genetic variant could be discussed with arguments a favor, but also on the contrary. At this point, it is important to remark that we cannot discard the existence of other genetic alterations in other genes, not known to date, as causes of PA-IVS. In consequence, several studies should be performed to corroborate this new association.

Finally, concerning potential therapeutic management, this patient will be followed both for his RVOT condition and his channelopathy. Normal follow-up in these children is performed using basal ECG every 6–12 months, trying to obtain an ECG during fever (to see the effect of temperature in his particular situation), and with the avoidance of sodium channel-blocking drugs (www.brugadadrugs.org) and monitoring with the implanted loop recorders (ILR) in children. In addition, ILR can help in two different situations: detecting asymptomatic arrhythmias and, most commonly, ruling out cardiac origin of unclear symptoms.

## 4. Conclusions

The present case raises, for the first time, new questions about pathogenic variants in the *SCN5A* gene as a cause of BrS concomitant to pulmonary atresia with an intact ventricular septum. This association suggests that deleterious variants in the *SCN5A* gene could be implicated in pulmonary atresia with an intact ventricular septum embryogenesis leading to overlapping phenotypes. It reinforces the need for an increasingly more integrated approach to children with CHD, with a high value on genetic studies and genotype–phenotype evaluation.

## Data Availability

The original contributions presented in the study are included in the article, further inquiries can be directed to the corresponding author.

## Abbreviations

CHD	congenital heart disease
PA-IVS	pulmonary atresia with intact ventricular septum
RVOT	right ventricular outflow tract
SCD	sudden cardiac death
